# Lost in the plot: missing visual elements in Kaplan-Meier plots of phase III oncology trials

**DOI:** 10.1093/oncolo/oyae067

**Published:** 2024-06-01

**Authors:** Alexander D Sherry, Pavlos Msaouel, Ramez Kouzy, Joseph Abi Jaoude, Timothy A Lin, Cullen M Taniguchi, Clifton David Fuller, Bruce Minsky, Ethan B Ludmir

**Affiliations:** Department of Radiation Oncology, Division of Radiation Oncology, The University of Texas MD Anderson Cancer Center, Houston, TX, United States; Department of Genitourinary Medical Oncology, Division of Cancer Medicine, The University of Texas MD Anderson Cancer Center, Houston, TX, United States; Department of Translational Molecular Pathology, Division of Pathology/Lab Medicine, The University of Texas MD Anderson Cancer Center, Houston, TX, United States; Department of Radiation Oncology, Division of Radiation Oncology, The University of Texas MD Anderson Cancer Center, Houston, TX, United States; Department of Radiation Oncology, Division of Radiation Oncology, The University of Texas MD Anderson Cancer Center, Houston, TX, United States; Department of Radiation Oncology, Stanford University, Stanford, CA, United States; Department of Radiation Oncology and Molecular Radiation Sciences, Johns Hopkins University School of Medicine, Baltimore, MD, United States; Department of Gastrointestinal Radiation Oncology, Division of Radiation Oncology, The University of Texas MD Anderson Cancer Center, Houston, TX, United States; Department of Experimental Radiation Oncology, Division of Radiation Oncology, The University of Texas MD Anderson Cancer Center, Houston, TX, United States; Department of Radiation Oncology, Division of Radiation Oncology, The University of Texas MD Anderson Cancer Center, Houston, TX, United States; Department of Gastrointestinal Radiation Oncology, Division of Radiation Oncology, The University of Texas MD Anderson Cancer Center, Houston, TX, United States; Department of Radiation Oncology, Division of Radiation Oncology, The University of Texas MD Anderson Cancer Center, Houston, TX, United States; Department of Gastrointestinal Radiation Oncology, Division of Radiation Oncology, The University of Texas MD Anderson Cancer Center, Houston, TX, United States; Department of Biostatistics, The University of Texas MD Anderson Cancer Center, Houston, TX, United States

**Keywords:** randomized controlled trials, phase III, Kaplan-Meier, clinical research, data visualization, time-to-event outcomes

## Abstract

Missing visual elements (MVE) in Kaplan-Meier (KM) curves can misrepresent data, preclude curve reconstruction, and hamper transparency. This study evaluated KM plots of phase III oncology trials. MVE were defined as an incomplete *y*-axis range or missing number at risk table in a KM curve. Surrogate endpoint KM curves were additionally evaluated for complete interpretability, defined by (1) reporting the number of censored patients and (2) correspondence of the disease assessment interval with the number at risk interval. Among 641 trials enrolling 518 235 patients, 116 trials (18%) had MVE in KM curves. Industry sponsorship, larger trials, and more recently published trials were correlated with lower odds of MVE. Only 3% of trials (15 of 574) published surrogate endpoint KM plots with complete interpretability. Improvements in the quality of KM curves of phase III oncology trials, particularly for surrogate endpoints, are needed for greater interpretability, reproducibility, and transparency in oncology research.

## Teaser

Missing visual elements in Kaplan-Meier (KM) curves can misrepresent data, preclude curve reconstruction, and hamper transparency. This study evaluated KM plots of phase III oncology trials.

**Implications for Practice**

The results of this study showed that most surrogate endpoint Kaplan-Meier (KM) plots were not fully interpretable. Improved adherence to quality guidelines for KM plots, particularly for trials evaluating surrogate endpoints, is needed to improve the interpretability, transparency, and reproducibility of phase III oncology research.

## Introduction

Kaplan-Meier (KM) curves are the most commonly used visual presentation of time-to-event outcomes in oncology; these plots rely on standard visual features for interpretability.^1,2^ Missing visual elements (MVE) in KM curves may distort data, mislead readers, and prevent secondary analyses.^3,4^ For example, in a recent study using survival curve reconstructions, Das et al^5^ excluded 66 of 405 phase III trials because of missing data in the KM plot. MVE may also prevent the assessment of key trial assumptions, such as proportional hazards or lack of informative censoring. Despite published guidelines, the quality of KM curves in contemporary trials remains unclear.^2,3^ Thus, this study was conducted to evaluate KM plots of published phase III oncology trials.

## Methods

**Figure 1.**
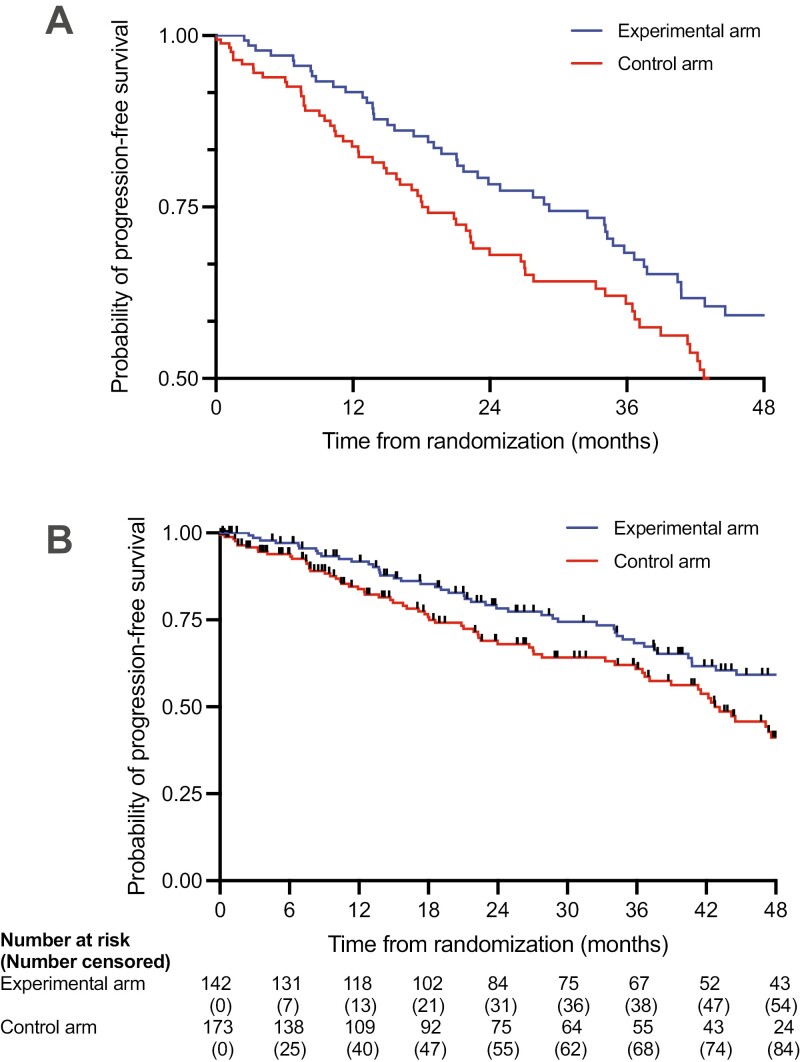
Example of Kaplan-Meier curves (A) with missing visual elements (MVE) and (B) without MVE. Data were fabricated using a random number generator for a hypothetical randomized controlled trial comparing progression-free survival between two groups, assessed every 6 months following 1:1 randomization; tick marks represent censoring. In (A), the *y*-axis is restricted to only survival probabilities between 0.50 and 1.00, which exaggerates the visual differences between groups and misrepresents the overall outcomes of the patients. In (B), the number at risk interval shows that the experimental group has fewer patients than the control group following 1:1 randomization. This scenario may occur in per-protocol analyses when an experimental therapy has considerable upfront toxicity, resulting in a systematic loss of patients compared with intention-to-treat analysis. The number at risk table in (B), which includes the number censored, also suggests informative censoring in the control arm at 6 months compared with the experimental arm (14% vs 5%), which may add bias in favor of the experimental arm.

Phase III oncology trials were screened from the ClinicalTrials.gov registry using previously reported search criteria.^6^ Institutional review board approval was not required. The study objective was to evaluate the incidence of any MVE in KM plots, defined as (1) an incomplete range for survival probability, or (2) missing number at risk (Figure 1).^1,3^ Surrogate endpoints were defined based on previously reported criteria.^6^ Because surrogate endpoints are influenced by the interval of disease assessments and potentially impacted by informative censoring, KM plots of surrogate endpoints were assessed for complete interpretability, which was defined by the following: (1) there were no MVE, (2) the number of patients censored (or number of events, from which the number censored could be derived) was reported over time, and (3) the number at risk interval corresponded to the assessment interval (Figure 1).^7^ If the assessment interval changed over time, the number at risk interval was considered corresponding if the intervals overlapped for at least half of the KM plot. Number at risk intervals that were more frequent than the assessment intervals were also considered corresponding, so long as the number at risk was reported at each assessment time point.

Trends were examined by ordinary least squares linear regression. Structural causal models were created for each trial characteristic to identify confounder variables (Supplementary Figure S1).^8^ Multivariable logistic regressions calculated adjusted odds ratios (aOR). All tests were 2-sided. Significance was set at 0.05, and CIs were calculated at 95%. Analyses were performed using SAS v9 (Cary, NC) and plots were created using Prism v9 (GraphPad, La Jolla, CA).

## Results

**Figure 2.**
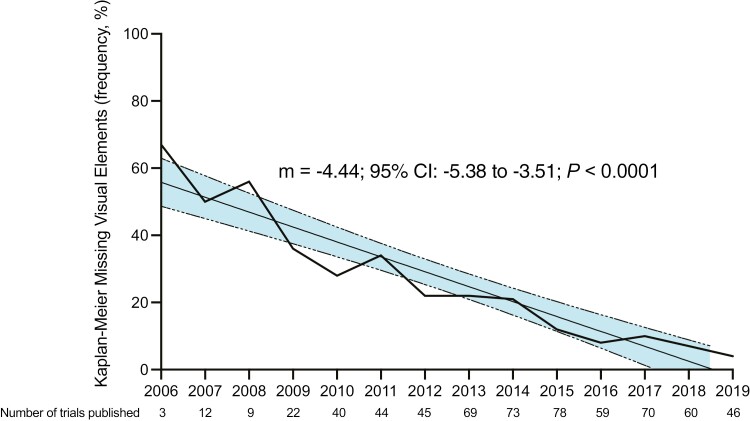
Trends in phase III oncology trials with missing visual elements in Kaplan-Meier plots over time. The linear regression over time is shown with the shaded regions representing the 95% CI of the slope. Because of the small number of trials analyzed in this dataset published in the years prior to 2006 (*N* = 5) or after 2019 (*N* = 6), data prior to 2006 or after 2019 were excluded from the graph.

Of 1877 screened trials, 1036 were phase III interventional randomized trials; of these, 395 were excluded (lack of manuscript, *N* = 251; lack of KM curve, *N* = 144), leaving a total of 641 trials enrolling 518 235 patients eligible, with publications from 2002 to 2020. Among these, 116 trials (18%) had MVE in KM curves (Supplementary Table S1). Specifically, 19 trials (3%) excluded the possible range(s) of survival probabilities, and 103 trials (16%) did not report the number at risk. MVE in surrogate endpoint KM plots and overall survival KM plots were found in 15% of trials (87/574; *y*-axis exclusions: 11/574; missing number at risk: 82/574) and 15% of trials (78/513; *y*-axis exclusions: 5/513; missing number at risk: 75/514), respectively. MVE decreased over time (m = −4.44; 95% CI: −5.38 to −3.51, *P* < 0.0001) from 45% (9/20, 2002-2007) to 34% (39/115, 2008-2011) to 18% (49/265, 2012-2015) to 8% (18/235, 2016-2019; *P* < 0.0001; Figure 2). High-impact journals publishing a plurality of phase III trials appeared to have lower rates of MVE: MVE incidence for trials published in *The Lancet* and *Lancet Oncology* was 4% (2/53) and 1% (1/109), respectively. MVE seemed to be associated with certain factors (Supplementary Table S2). On adjusted analysis, trials studying metastatic solid tumors, trials with industry funding, more recently published trials, and larger trials were associated with lower odds of MVE (Supplementary Table S3). The association of enrollment and MVE persisted when evaluating trials with enrollments exceeding 200 patients as well as trials with enrollments exceeding 100 patients, suggesting this overall association is attributable to a few small trials (Supplementary Table S4).

Only 3% of trials (15/574) displayed surrogate endpoint KM plots with complete interpretability. The number of censored patients over time was present in 9% of trials (50/574), and the disease assessment interval (when reported) corresponded with the number at risk interval in 27% of trials (139/507). All trials with completely interpretable surrogate endpoint KM plots were published in either a *Lancet* group journal (*N* = 13) or the *New England Journal of Medicine* (*N* = 2).

## Discussion

Our study demonstrates a modest prevalence of MVE in KM plots of phase III oncology trials. The rate of MVE overall has reassuringly declined over time. Trial-level factors, including publication journal, enrollment, and sponsor, appear associated with lower rates of MVE, which may be related to methodological rigor and quality editorial review. However, only 3% of phase III trials published completely interpretable surrogate endpoint KM plots. Trials evaluating surrogate endpoints should report the number at risk plus the number of censored patients over time. The number at risk interval should correspond to the interval of disease assessment for full interpretability of the study’s findings and assessment of key assumptions, such as informative censoring.^7,9^

Future trials should consider the novel strategy of displaying 95% CIs for the difference in outcomes, which may better facilitate visual comparative inferences compared to plotting the curves alone. 95% CIs for group-specific outcomes are not recommended for convenience samples typical in randomized trials.^10^

Limitations of this study include the source database (ClinicalTrials.gov), which may not be representative of global trials. KM curves in the supplement section of manuscripts were not assessed, and this may have reduced the detection of MVE.

In summary, there is a modest and decreasing prevalence of MVE in the KM plots of phase III oncology trials. However, the vast majority of surrogate endpoint KM plots were not fully interpretable. Improved adherence to quality guidelines for KM plots, particularly for trials evaluating surrogate endpoints, is needed to improve the interpretability, transparency, and reproducibility of phase III oncology research.

## Supplementary Material

oyae067_suppl_Supplementary_Material

## Data Availability

Research data are stored in an institutional repository and will be shared upon reasonable request to the corresponding author for up to one year following publication.
