# Supplementary material for: Lost in the plot: missing visual elements in Kaplan-Meier plots of phase III oncology trials
Source: Oncologist. 2024 Jun 1;29(7):547–50. doi: 10.1093/oncolo/oyae067 (PMC11224967; doi:10.1093/oncolo/oyae067)
Supplement: oyae067_suppl_Supplementary_Material [file oyae067_suppl_supplementary_material.pdf]

Supplementary Online Content

Lost in the Plot: Missing Visual Elements in Kaplan-Meier Plots of Phase III Oncology Trials

Figure S1. Example structural causal model depicted on a directed acyclic graph.

Table S1. Characteristics of trials and frequency of Kaplan-Meier missing visual element.

Table S2. Unadjusted regressions of trial-level characteristics and missing visual elements.

Table S3. Adjusted regressions of trial-level characteristics and missing visual elements.

Table S4. Adjusted regressions of enrollment using different cutoffs and missing visual elements.

Figure S1. An example structural causal model depicted on a directed acyclic graph illustrating the putative relationships between trial-level characteristics and missing visual elements (MVE). In this example, publication year is the factor of interest (shown in green). The outcome (MVE) is shown in blue. Confounders specific to the relationship between publication year and MVE are treatment type and enrollment size (shown in yellow). These confounders were included in the multivariable model evaluating the effect of publication year on MVE.

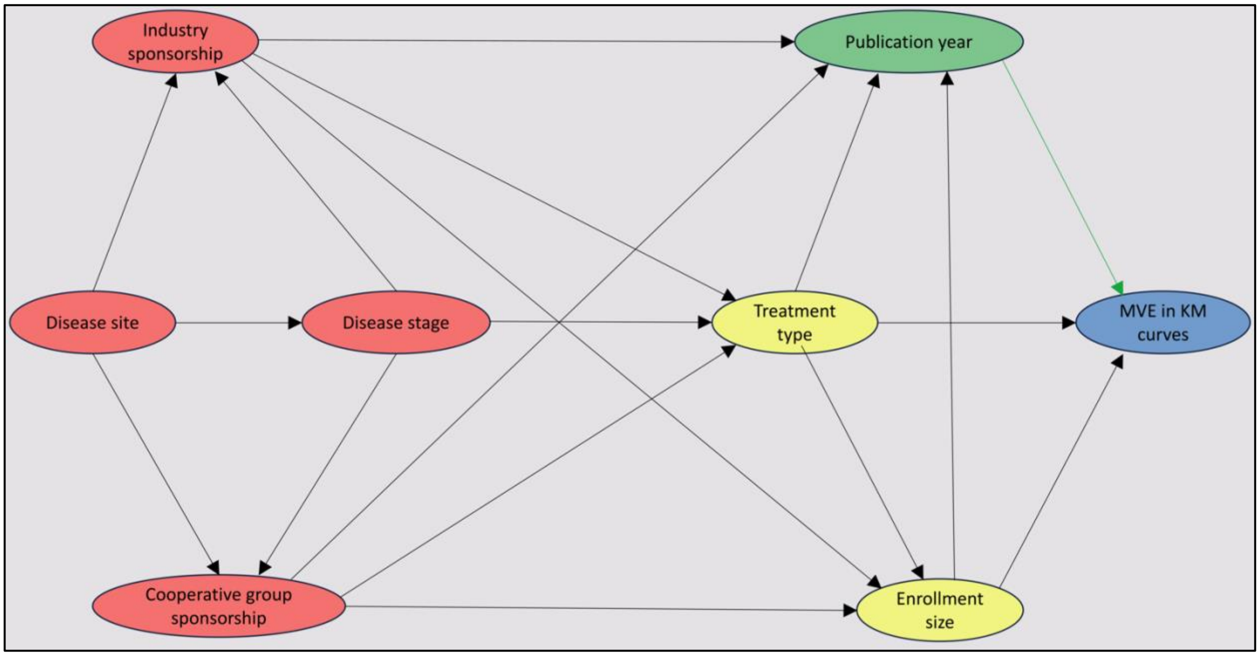

Table S1. Characteristics of phase III oncology randomized controlled trials and Kaplan-Meier plots with missing visual elements (MVE).

Characteristic	All trials, N	Kaplan-Meier MVE, N (%)
**Total trials**	641	116 (18%)
**Disease Stage**		
Solid M0	138	32 (23%)
Solid M1	374	47 (13%)
Hematologic	129	37 (29%)
**Disease Site**		
Breast	112	14 (13%)
Gastrointestinal	91	14 (15%)
Genitourinary	79	12 (15%)
Hematologic	129	37 (29%)
Thoracic	107	15 (14%)
Other[a]	123	24 (20%)
**Treatment Type**		
Supportive Care	40	24 (60%)
Local Therapy	23	6 (26%)
Systemic Therapy	578	86 (15%)
**Cooperative Study**	182	57 (31%)
**Industry Funded**	513	65 (13%)
**Enrollment[b]**		
<323	160	52 (33%)
<563	159	20 (13%)
<889	162	23 (14%)
≥889	160	21 (13%)

Abbreviations: M0 = non-metastatic; M1 = metastatic.

[a] Includes central nervous system, skin, endocrine, gynecologic, sarcoma, pediatric, head and neck, and trials evaluating multiple disease sites.

[b] Enrolled number of patients is categorized by quartiles.

Table S2. Unadjusted associations of phase III oncology randomized controlled trial characteristics and Kaplan-Meier plots with missing visual elements (MVE).

Characteristic	OR	95% CI	P
**Disease Stage**			
Solid M0	0.75	0.43 to 1.30	0.31
Solid M1	0.36	0.22 to 0.58	<0.0001
Hematologic	Ref		
**Disease Site**			
Breast	0.59	0.28 to 1.19	0.15
Gastrointestinal	0.75	0.36 to 1.53	0.44
Genitourinary	0.74	0.34 to 1.55	0.44
Hematologic	1.66	0.93 to 3.01	0.09
Thoracic	0.67	0.33 to 1.35	0.27
Other[a]	Ref		
**Treatment Type**			
Supportive Care	8.58	4.42 to 17.11	<0.0001
Local Therapy	2.02	0.71 to 5.01	0.15
Systemic Therapy	Ref		
**Cooperative Study**	3.09	2.04 to 4.69	<0.0001
**Industry Funded**	0.22	0.14 to 0.34	<0.0001
**Enrollment**[b]			
<323	Ref		
<563	0.30	0.16 to 0.52	<0.0001
<889	0.34	0.20 to 0.59	0.0001
≥889	0.31	0.18 to 0.59	<0.0001
**Publication Year**	0.81	0.76 to 0.86	<0.0001

Abbreviations: M0 = non-metastatic; M1 = metastatic; OR = odds ratio.

[a] Includes central nervous system, skin, endocrine, gynecologic, sarcoma, pediatric, head and neck, and trials evaluating multiple disease sites.

[b] Enrolled number of patients is categorized by quartiles and analyzed as an un-ordered categorical variable.

Table S3. Adjusted associations of phase III oncology randomized controlled trial characteristics and Kaplan-Meier plots with missing visual elements (MVE). Only the effect estimates from the factor of interest for each multivariable model are shown here. Confounder variables included in the models informed by directed acyclic graphs and their effect estimates are not displayed.

Characteristic	aOR	95% CI	P
**Disease Stage**			
Solid M0	0.97	0.50 to 1.86	0.92
Solid M1	0.45	0.24 to 0.84	0.01
Hematologic	Ref		
**Disease Site**			
Breast	0.59	0.28 to 1.19	0.15
Gastrointestinal	0.75	0.36 to 1.53	0.44
Genitourinary	0.74	0.34 to 1.55	0.44
Hematologic	1.66	0.93 to 3.01	0.09
Thoracic	0.67	0.33 to 1.35	0.27
Other[a]	Ref		
**Treatment Type**			
Supportive Care	5.83	2.88 to 12.02	<0.0001
Local Therapy	0.80	0.27 to 2.11	0.66
Systemic Therapy	Ref		
**Cooperative Study**	1.39	0.76 to 2.49	0.27
**Industry Funded**	0.29	0.15 to 0.53	<0.0001
**Enrollment[b]**			
<323	Ref		
<563	0.38	0.20 to 0.69	0.002
<889	0.45	0.25 to 0.80	0.007
≥889	0.36	0.20 to 0.66	0.0009
**Publication Year**	0.79	0.74 to 0.85	<0.0001

Abbreviations: M0 = non-metastatic; M1 = metastatic; aOR = adjusted odds ratio.

[a] Includes central nervous system, skin, endocrine, gynecologic, sarcoma, pediatric, head and neck, and trials evaluating multiple disease sites.

[b] Enrolled number of patients is categorized by quartiles and analyzed as an un-ordered categorical variable.

Table S4. Adjusted associations of phase III oncology randomized controlled trial characteristics and Kaplan-Meier plots with missing visual elements (MVE) evaluating different approaches to analyzing the enrollment variable.

Characteristic	aOR	95% CI	P
**Enrollment**			
<100	Ref		
≥100	0.30	0.11 to 0.86	0.02
**Enrollment**			
<200	Ref		
≥200	0.23	0.13 to 0.41	<0.0001
